# Importance of Patient History in Artificial Intelligence–Assisted Medical Diagnosis: Comparison Study

**DOI:** 10.2196/52674

**Published:** 2024-04-08

**Authors:** Fumitoshi Fukuzawa, Yasutaka Yanagita, Daiki Yokokawa, Shun Uchida, Shiho Yamashita, Yu Li, Kiyoshi Shikino, Tomoko Tsukamoto, Kazutaka Noda, Takanori Uehara, Masatomi Ikusaka

**Affiliations:** 1 Department of General Medicine, Chiba University Hospital, Chiba-shi, Japan; 2 Uchida Internal Medicine Clinic, Saitama-shi, Japan

**Keywords:** medical diagnosis, ChatGPT, AI in medicine, diagnostic accuracy, patient history, medical history, artificial intelligence, AI, physical examination, physical examinations, laboratory investigation, laboratory investigations, mHealth, accuracy, public health, United States, AI diagnosis, treatment, male, female, child, children, youth, adolescent, adolescents, teen, teens, teenager, teenagers, older adult, older adults, elder, elderly, older person, older people, investigative, mobile health, digital health

## Abstract

**Background:**

Medical history contributes approximately 80% to a diagnosis, although physical examinations and laboratory investigations increase a physician’s confidence in the medical diagnosis. The concept of artificial intelligence (AI) was first proposed more than 70 years ago. Recently, its role in various fields of medicine has grown remarkably. However, no studies have evaluated the importance of patient history in AI-assisted medical diagnosis.

**Objective:**

This study explored the contribution of patient history to AI-assisted medical diagnoses and assessed the accuracy of ChatGPT in reaching a clinical diagnosis based on the medical history provided.

**Methods:**

Using clinical vignettes of 30 cases identified in *The BMJ*, we evaluated the accuracy of diagnoses generated by ChatGPT. We compared the diagnoses made by ChatGPT based solely on medical history with the correct diagnoses. We also compared the diagnoses made by ChatGPT after incorporating additional physical examination findings and laboratory data alongside history with the correct diagnoses.

**Results:**

ChatGPT accurately diagnosed 76.6% (23/30) of the cases with only the medical history, consistent with previous research targeting physicians. We also found that this rate was 93.3% (28/30) when additional information was included.

**Conclusions:**

Although adding additional information improves diagnostic accuracy, patient history remains a significant factor in AI-assisted medical diagnosis. Thus, when using AI in medical diagnosis, it is crucial to include pertinent and correct patient histories for an accurate diagnosis. Our findings emphasize the continued significance of patient history in clinical diagnoses in this age and highlight the need for its integration into AI-assisted medical diagnosis systems.

## Introduction

Over the past decade, medical knowledge and diagnostic techniques have expanded globally and have become more accessible with remarkable advancements in clinical testing and useful reference systems. Despite these advancements, misdiagnosis significantly contributes to mortality, making it a significant public health issue [1,2]. Studies have shown discrepancies between clinical and postmortem autopsy diagnoses in at least 25% of patients [3-7]. One study suggests that approximately 40,500 adult patients in intensive care units in the United States die of misdiagnoses annually, and the predicted prevalence of potentially lethal misdiagnoses is 6.3% [8]. Another report suggests that diagnostic errors contribute to approximately 10% of deaths and 6% to 17% of hospital adverse events, and are the leading cause of medical malpractice claims [7]. Considering the operative characteristics of clinical investigations combined with the inherent variability in disease presentation, it is often challenging to diagnose patients correctly—an issue that has concerned physicians perennially. Decades ago, a pivotal study proposed that patient history contributes to approximately 80% of the diagnostic process [9,10]. Medical history remains crucial for diagnosis [11,12] and is vital in contemporary physicians’ clinical diagnoses.

With the advent of artificial intelligence (AI) in recent years, numerous studies have focused on AI-assisted diagnoses, including cancer screening and treatment [13-15], diagnostic ultrasound imaging [16-19], x-ray imaging [20], computed tomography [21], magnetic resonance imaging [22], and endoscopy [15,23]. Other reports on AI-assisted imaging diagnoses include AI’s applications in radiology, pathology, and dermatological imaging [13,24]. There have also been reports on the use of AI in diagnosing specific conditions [25-27]. However, while several studies have reported that AI is useful in screening, diagnosing, and even treating certain medical conditions, to the best of our knowledge, no study has examined the importance of patient history in AI-assisted medical diagnosis. In addition, the extent to which AI considers patient history in its diagnostic processes remains to be fully understood.

This study aimed to investigate the importance of patient history in an AI-assisted medical diagnostic process aided by ChatGPT (version 4.0; June 2, 2023), one of the most well-known large language models that was released on March 14, 2023, to better understand the future of diagnostic medicine where AI is predicted to play an increasingly prominent role. Our study explored the contribution of patient history to AI-assisted medical diagnoses and assessed the accuracy of ChatGPT in reaching a clinical diagnosis based on the medical history that was provided. By reevaluating the significance of patient history, our study contributes to the ongoing discourse on optimizing diagnostic processes, both conventional and AI-assisted.

## Methods

### Study Design, Settings, and Participants

**Figure 1.**
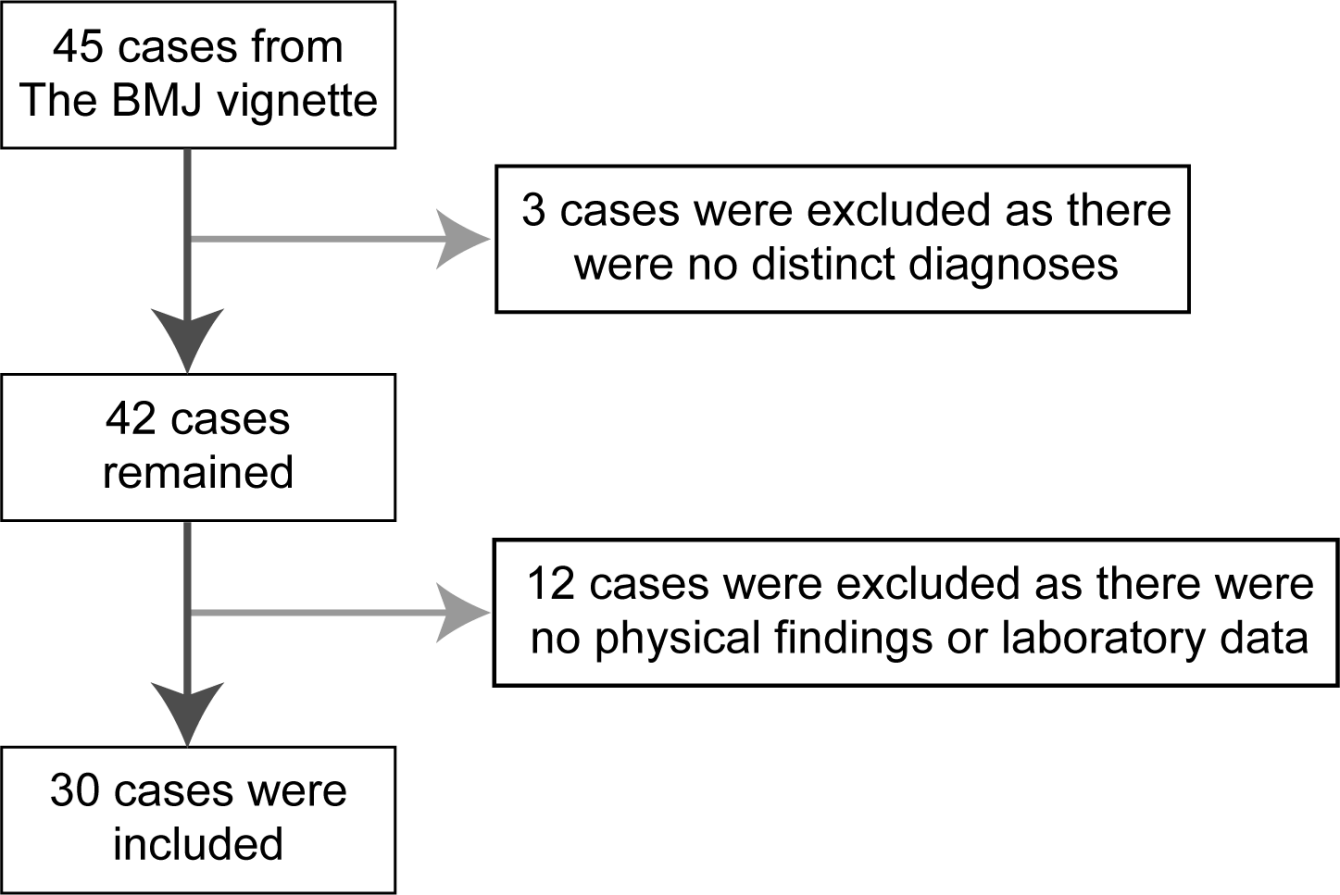
Inclusion and exclusion criteria.

In our study, we used some of the 45 standardized clinical vignettes in *The BMJ* (Multimedia Appendix 1) to evaluate the diagnostic and triage accuracy of web-based symptom checkers [28]. These vignettes were published on June 5, 2015. They offer a balanced set of cases, with 15 cases requiring immediate attention, 15 cases requiring consultation but not immediately, and 15 cases not requiring immediate attention or consultation. They were identified from various clinical sources, including materials used to educate health professionals as well as a medical resource website, with content provided by a panel of physicians. Researchers have used these clinical vignettes to evaluate the usefulness of web-based symptom checkers and self-triage [28-31]. We chose these vignettes because of their varied severity levels, their origins from multiple resources rather than just 1 resource, and their credibility, having been used in prior studies. They also include some of the most commonly observed conditions in outpatient settings. Of the 45 cases, we selected those that included physical examination findings, test data, and medical history and provided a single distinct diagnosis. As illustrated in Figure 1, we excluded patients with no distinct diagnoses within the vignettes to serve as a reference (3 cases) and those who did not undergo any physical examination or laboratory tests (12 cases). Finally, the remaining 30 cases were used in this study.

### Data Collection and Measurements

We assigned the correct diagnosis for each of these 30 cases to “Answer.” We then used the AI model, ChatGPT, to generate 2 diagnoses: the first, labeled “History,” was obtained by inputting only the medical history into ChatGPT; the second set, labeled “All,” was produced by inputting the medical history and all the other additional information in the clinical vignettes. Each time ChatGPT was prompted to generate a diagnosis, a separate chat window was used (Multimedia Appendix 2). Thus, we used 2 chat windows for each case—one for the “History” diagnosis and the other for the “All” diagnosis. Additionally, the patients’ information was not inputted incrementally.

The concordance rate was assessed among “Answer,” “History,” and “All.” To extract a diagnosis from ChatGPT, we ended each input session with the phrase “What is the most likely diagnosis?” For both the “History” and “All,” the session was deemed complete when the AI returned the single most likely diagnosis. If ChatGPT suggested multiple diagnoses or indicated that it did not provide the most likely diagnosis, we repeated the process under the same conditions for a maximum of 5 attempts. Cases for which a single diagnosis could not be obtained even after 5 attempts were excluded without making further attempts.

### Ethical Considerations

Our research does not involve humans, medical records, patient information, observations of public behaviors, or secondary data analyses; hence, it is exempt from ethical approval, the requirement of informed consent, and institutional review board approval. Additionally, as no identifying information was included, the data did not need to be anonymized or deidentified, and the need for compensation did not arise because no human participants were included in the study.

### Data Analysis

Three board-certified physicians working in a medical diagnostic department at our facility assessed the concordance among the 3 AI-proposed diagnoses (“Answer,” “History,” and “All”). Of the 3 physicians, 1 is general medicine board–certified, 1 is internal medicine board–certified, and 1 is internal medicine–, general internal medicine–, and family medicine board–certified; their postgraduate education spanned 7, 9, and 11 years, respectively. A diagnosis was considered to match if at least 2 of the 3 physicians agreed upon the correspondence. Distinguishing between acute pharyngitis and acute upper respiratory tract infection necessitated determining whether to consider diseases resulting from similar pathologies as correct diagnoses. In contrast, for diseases that are essentially the same but have different nomenclatures, such as oral ulcers and canker sores, we considered them correct diagnoses.

## Results

Among the 30 cases, 19 patients were male and 11 were female, with ages ranging from 18 months to 65 years. In total, 12 individuals were younger than 20 years.

The results are shown in Table 1. Cases 1-15 of the original vignette represent those requiring emergent care, cases 16-30 represent those requiring nonemergent care, and cases 31-45 represent those that are appropriate for self-care. A comparison with the correct diagnosis listed in *The BMJ* vignettes (labeled as “Answer”) showed that “Answer” and “History” coincided 76.6% of the time, while “Answer” and “All” had a concordance rate of 93.3%. Five (16.7%) patients could not be diagnosed on the basis of medical history alone but were diagnosed when additional information was provided. In 1 (3.3%) case, the diagnosis was different and incorrect under both conditions (“History” and “All”). In 1 (3.3%) case, the incorrect diagnosis was the same under both conditions (“History” and “All”).

**Table 1.** List of answers and diagnoses made by ChatGPT^a^.

Case number of the original vignette	Original diagnosis (Answer)	Output from history only (History)^b^	Output from all information (All)^c^
1	Acute liver failure	Acute liver failure^d^	Acute liver failure^d^
2	Appendicitis	Acute gastroenteritis	Acute peritonitis, possibly secondary to a ruptured appendix (perforated appendicitis)^d^
5	Deep vein thrombosis	Deep vein thrombosis^d^	Deep vein thrombosis^d^
6	Heart attack	Acute myocardial infarction^d^	Acute anterior wall myocardial infarction^d^
7	Hemolytic uremic syndrome	Hemolytic uremic syndrome^d^	Hemolytic uremic syndrome^d^
9	Malaria	Malaria^d^	Malaria^d^
10	Meningitis	N/A^e^ × 5^f^	Meningitis^d^
11	Pneumonia	Community-acquired pneumonia^d^	Community-acquired pneumonia^d^
12	Pulmonary embolism	Pulmonary embolism^d^	Pulmonary embolism^d^
13	Rocky Mountain spotted fever	Tick-borne illness, such as Rocky Mountain spotted fever or ehrlichiosis^d^	Rocky Mountain spotted fever^d^
16	Acute otitis media	Viral upper respiratory tract infection	Acute otitis media^d^
17	Acute pharyngitis	Strep throat^d^	Streptococcal pharyngitis^d^
18	Acute pharyngitis	Streptococcal pharyngitis^d^	Streptococcal pharyngitis^d^
19	Acute sinusitis	Acute sinusitis^d^	N/A × 2^g^; acute bacterial sinusitis^d^
21	Cellulitis	N/A × 5	Cellulitis^d^
24	Mononucleosis	Infectious mononucleosis^d^	Infectious mononucleosis^d^
25	Peptic ulcer disease	Peptic ulcer disease^d^	Peptic ulcer disease^d^
26	Pneumonia	Pneumonia^d^	Community-acquired pneumonia^d^
27	*Salmonella* infection	*Campylobacter jejuni* infection	Acute gastroenteritis, likely due to food poisoning
30	Vertigo	Benign paroxysmal positional vertigo^d^	Benign paroxysmal positional vertigo^d^
31	Acute bronchitis	Acute bronchitis^d^	Acute bronchitis^d^
32	Acute bronchitis	Acute bronchitis^d^	Acute bronchitis^d^
33	Acute conjunctivitis	Viral conjunctivitis^d^	Viral conjunctivitis^d^
34	Acute pharyngitis	Viral upper respiratory tract infection	Upper respiratory tract infection
37	Bee sting without anaphylaxis	Pain of the sting	Localized allergic reaction to a bee sting^d^
38	Canker sore	Recurrent aphthous stomatitis^d^	Recurrent aphthous stomatitis^d^
39	Candida yeast infection	Vaginal candidiasis^d^	Vulvovaginal candidiasis^d^
42	Stye	Hordeolum^d^	Hordeolum^d^
43	Viral upper respiratory tract infection	Acute sinusitis^d^	Acute sinusitis^d^
44	Viral upper respiratory tract infection	Common viral illness, such as the common cold or influenza^d^	Viral upper respiratory tract infection^d^

a We repeated outputs until a single plausible diagnosis was made, with a maximum of 5 attempts.

b Matching answers between Answer and History: 23/30 (76.6%); median trial count 1 (Q1 1, Q2 1, Q3 1).

c Matching answers between History and All: 28/30 (93.3%); median trial count 1 (Q1 1, Q2 1, Q3 1).

d The output matched with that of “Answer.”

e N/A: not applicable.

f We attempted to obtain a diagnosis 5 times but failed.

g We attempted to obtain a diagnosis twice but failed.

**Figure 2.**
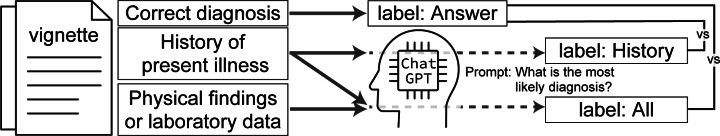
Data collection and measurements.

Figure 2 presents details regarding the number of attempts required. On average, 1.27 attempts were needed for inputs involving only medical history followed by the question “What is the most likely diagnosis?” When all possible information, including physical examination findings and laboratory data, were inputted, followed by the same question, an average of 1.00 attempt was required. Regarding the 2 cases shown in Figure 2 that required 5 attempts, ChatGPT was unable to narrow down the diagnosis to the single most likely option. Consequently, these cases were counted as mismatches with the correct diagnoses listed in *The BMJ* vignettes.

## Discussion

### Principal Findings

Despite the advancements in medical knowledge and diagnostic techniques, misdiagnosis remains a significant issue. AI has shown promise in the diagnosis and treatment of medical conditions; however, there is limited understanding of how AI uses patient history for diagnostic purposes. Our study aimed to investigate the extent to which AI (ChatGPT) can use information from medical history to accurately diagnose common diseases, which are frequently encountered in general outpatient, emergency, and ward management settings. Although some studies have investigated the accuracy of AI-based medical diagnosis, our study is novel because it emphasizes the importance of patient history. We compared the diagnostic accuracy of diagnoses made on the basis of only patient history and those made using all the information; this makes our study unique. To the best of our knowledge, no previous research has been conducted on this topic.

Our study investigated the role of patient history in AI-assisted medical diagnoses using ChatGPT. We analyzed 30 standardized patient vignettes from *The BMJ* to assess the concordance rates between AI-proposed diagnoses based on medical history only and those based on both medical history and additional information. Our results showed high concordance rates of 76.6% between the “Answer” and “History” groups, suggesting the importance of patient history in AI-assisted diagnoses and highlighting the potential of AI in improving diagnostic accuracy. This result is similar to that of a previous study that involved actual physicians instead of ChatGPT [9,10].

Characteristics of cases that did not lead to appropriate diagnoses based on history alone include, for instance, the following: an appendicitis case (case 2 in Multimedia Appendix 1) for which there was no documentation of pain migration in the medical history, a meningitis case (case 10 in Multimedia Appendix 1) wherein only headache and fever were documented, an otitis media case (case 16 in Multimedia Appendix 1) wherein only upper respiratory symptoms were recorded with no mention of ear-related symptoms, errors in identifying the causative agent in a case of acute gastroenteritis (case 27 in Multimedia Appendix 1), and an acute pharyngitis case (case 34 in Multimedia Appendix 1) that lacked the necessary medical history to determine the Centor score. Such omissions in the medical history could be considered contributing factors to the misdiagnoses. When physical findings and test data were added, an accurate diagnosis was achieved in 28 out of 30 cases (93.3%), showing a 16.7% increase in the accuracy rate. These two cases were of acute pharyngitis diagnosed as acute upper respiratory tract infection and *Salmonella* enteritis diagnosed as acute gastroenteritis. While we considered these incorrect diagnoses for the purpose of this study, they could have been deemed correct under certain criteria. Of the 7 cases that did not match between “Answer” and “History,” 6 were of infectious diseases (21 of 30 cases were of infectious diseases). These included cases where appendicitis was mistaken for acute gastroenteritis, acute otitis media and acute pharyngitis were mistaken for upper respiratory infections, and a *Salmonella* infection was mistaken for a *Campylobacter* infection. Physical examinations or tests may help identify the site of infection or pathogen in cases of intra-abdominal or head and neck infections.

There are situations in which physical examination and clinical test information may not be available in clinical settings. For instance, digital patient encounters owing to the impact of the COVID-19 pandemic often preclude physical examinations and clinical tests. The widespread use of telemedicine approaches in COVID-19 management, from screening to follow-up, has demonstrated the community’s acceptance and interest in telehealth solutions [32]. Moreover, even in face-to-face consultations, there are scenarios, such as in clinics, where detailed clinical tests may not be feasible depending on the setting. Furthermore, we cannot perform all physical examinations and tests on all patients. Therefore, we should consider potential differential diagnoses and decide which pertinent physical examinations or tests are the most suitable and should be performed. Most importantly, it has been reported that one rarely makes a correct diagnosis when one cannot make a differential diagnosis based on history [11]. In addition, accurately predicting the diagnosis based on medical history is associated with a higher diagnostic accuracy of the physical examination, whereas incorrect prediction of the diagnosis based on medical history is associated with a lower diagnostic accuracy of the physical examination [33]. Based on these findings and suggestions, medical diagnosis using ChatGPT is considered heavily dependent on history.

Using AI for diagnosis can enhance diagnostic accuracy by more efficiently collecting medical histories. For instance, diagnosing acute appendicitis is sometimes challenging. AI may face the same challenge as that observed when, in our study, AI mistakenly identified acute appendicitis as acute gastroenteritis. This misdiagnosis may have occurred because the case lacked specific medical histories characteristic of appendicitis, such as pain migration. By configuring AI systems to verify pain migration in patients with abdominal pain, especially for such common conditions, diagnostic precision may improve.

There are 2 possible limitations in our study. First, it remains unclear whether similar results could be obtained with other vignettes or actual patients. Unlike using preprovided vignettes, among which we included 30 cases, diagnosis can be more challenging in clinical settings because it requires taking a medical history from patients. We included 30 cases from among the vignettes, which include some of the most commonly observed conditions in the outpatient setting. Although covering all the existing conditions is not feasible, we do not know if the case volume in our study is sufficiently high. This study included relatively simple cases in which patients had very few comorbidities, potentially making the diagnosis less challenging. Moreover, patients with psychiatric conditions tend to present with complex and lengthy case histories, and the wording used by mental health clinicians may differ, be inconsistent, be vague, or fail to pinpoint a diagnosis. Our vignettes did not include a diagnosis of any mental illness. Due to the abovementioned reasons, our results may not apply to all clinical settings. Furthermore, when we consider what the patient reports, results may differ if languages other than English are used since ChatGPT does not recognize some languages, and each language may have its unique nuance. This highlights the importance of linguistic diversity and cultural context in AI applications, particularly in medical diagnoses where patient communication and history are critical. Future iterations of AI systems should aim to incorporate a broader range of languages and understand cultural nuances to ensure more accurate and inclusive diagnostic support. This idea is important in the context of health inequality. Furthermore, disparities in technology access may pose some challenges. Future research should address these barriers to ensure equitable access to AI-assisted diagnostic tools.

Second, we encountered cases where the input of medical history followed by the question, “What is the most likely diagnosis?” failed to yield a single most likely diagnosis even after 5 attempts, which could have introduced bias into our results, although we only had 2 such cases.

In the future, studies should focus on training AI by implementing evidence-based medical information, enabling it to present the underlying reasons and guidelines for diagnoses. In the event of a misdiagnosis, analyzing the process that led to the false diagnosis could be challenging in an AI-assisted medical diagnosis. Given the current situation where reflection on misdiagnoses is not always feasible, AI should be used as an auxiliary tool in medical diagnosis. This approach underscores the importance of AI, deeming it a support system rather than a definitive diagnostic solution. This area needs further investigation. Future studies should also verify our results with certain common conditions or diseases, such as the top 10 diseases identified in the Global Burden of Diseases study [34], potentially leveraging the benefits and limitations of AI-assisted medical diagnosis.

### Conclusions

Relevant patient history is essential for AI-assisted diagnosis. The input of relevant patient history or the development of AI systems capable of obtaining comprehensive medical histories is vital for AI-assisted medical diagnosis. Furthermore, even in the modern era of advanced medical knowledge and clinical testing, the significance of patient history in diagnosis remains crucial.

## Supplementary material

10.2196/52674 Multimedia Appendix 1 Clinical Vignettes used in our study.

10.2196/52674 Multimedia Appendix 2 Explanation of the prompts we used in our study.

## Abbreviations

AI: artificial intelligence
