# Supplementary material for: Importance of Patient History in Artificial Intelligence–Assisted Medical Diagnosis: Comparison Study
Source: JMIR Med Educ. 2024 Apr 8;10:e52674. doi: 10.2196/52674 (PMC11024399; doi:10.2196/52674)
Supplement: Multimedia Appendix 2 [file mededu-v10-e52674-s002.pdf]

We have added the prompt “What is the most likely diagnosis” at the end of the patient information.

For example, when we evaluated Vignette No. 1,

We inputted “A 48-year-old woman with a history of migraine headaches presents to the emergency room with altered mental status over the last several hours. She was found by her husband, earlier in the day, to be acutely disoriented and increasingly somnolent. Her husband reports that she has consistently been taking pain medications and started taking additional 500 mg acetaminophen pills several days ago for lower back pain. Further history reveals a medication list with multiple acetaminophen-containing preparations. What is the most likely diagnosis?” into Chat GPT to yield “Output from Hx only” diagnosis.

We inputted “A 48-year-old woman with a history of migraine headaches presents to the emergency room with altered mental status over the last several hours. She was found by her husband, earlier in the day, to be acutely disoriented and increasingly somnolent. On physical examination, she has scleral icterus, mild right upper quadrant tenderness, and asterixis. Preliminary laboratory studies are notable for a serum ALT of 6498 units/L, total bilirubin of 5.6 mg/dL, and INR of 6.8. Her husband reports that she has consistently been taking pain medications and started taking additional 500 mg acetaminophen pills several days ago for lower back pain. Further history reveals a medication list with multiple acetaminophen-containing preparations. What is the most likely diagnosis?” to Chat GPT to yield “Output from all text” diagnosis.
