# Supplementary material for: Importance of Patient History in Artificial Intelligence–Assisted Medical Diagnosis: Comparison Study
Source: JMIR Med Educ. 2024 Apr 8;10:e52674. doi: 10.2196/52674 (PMC11024399; doi:10.2196/52674)
Supplement: Multimedia Appendix 1 [file mededu-v10-e52674-s001.pdf]

ID	Diagnosis	Patient information	History of present illness	Physical findings	Laboratory data
1	Acute liver failure	A 48-year-old woman with a history of migraine headaches presents to the emergency room with altered mental status over the last several hours. She was found by her husband, earlier in the day, to be acutely disoriented and increasingly somnolent. On physical examination, she has scleral icterus, mild right upper quadrant tenderness, and asterixis. Preliminary laboratory studies are notable for a serum ALT of 6498 units/L, total bilirubin of 5.6 mg/dL, and INR of 6.8. Her husband reports that she has consistently been taking pain medications and started taking additional 500 mg acetaminophen pills several days ago for lower back pain. Further history reveals a medication list with multiple acetaminophen-containing preparations.	A 48-year-old woman with a history of migraine headaches presents to the emergency room with altered mental status over the last several hours. She was found by her husband, earlier in the day, to be acutely disoriented and increasingly somnolent.<br>Her husband reports that she has consistently been taking pain medications and started taking additional 500 mg acetaminophen pills several days ago for lower back pain. Further history reveals a medication list with multiple acetaminophen-containing preparations.	On physical examination, she has scleral icterus, mild right upper quadrant tenderness, and asterixis.	Preliminary laboratory studies are notable for a serum ALT of 6498 units/L, total bilirubin of 5.6 mg/dL, and INR of 6.8.
2	Appendicitis	A 12-year-old girl presents with sudden-onset severe generalized abdominal pain associated with nausea, vomiting, and diarrhea. On exam she appears ill and has a temperature of 104°F (40°C). Her abdomen is tense with generalized tenderness and guarding. No bowel sounds are present.	A 12-year-old girl presents with sudden-onset severe generalized abdominal pain associated with nausea, vomiting, and diarrhea.	On exam she appears ill and has a temperature of 104°F (40°C). Her abdomen is tense with generalized tenderness and guarding. No bowel sounds are present.	-
3	Asthma	A 27-year-old woman with a history of moderate persistent asthma presents to the emergency room with progressive worsening of shortness of breath, wheezing, and cough over 3 days. She reports prior exposure to a person who had a runny nose and a hacking cough. She did not receive significant relief from her rescue inhaler with worsening symptoms, despite increased use. She has been compliant with her maintenance asthma regimen, which consists of an inhaled corticosteroid and a leukotriene receptor antagonist for maintenance therapy and albuterol as rescue therapy. Her cough is disrupting her sleep pattern and as a consequence she is experiencing daytime somnolence, which is affecting her job performance.	A 27-year-old woman with a history of moderate persistent asthma presents to the emergency room with progressive worsening of shortness of breath, wheezing, and cough over 3 days. She reports prior exposure to a person who had a runny nose and a hacking cough. She did not receive significant relief from her rescue inhaler with worsening symptoms, despite increased use. She has been compliant with her maintenance asthma regimen, which consists of an inhaled corticosteroid and a leukotriene receptor antagonist for maintenance therapy and albuterol as rescue therapy. Her cough is disrupting her sleep pattern and as a consequence she is experiencing daytime somnolence, which is affecting her job performance.	-	-
4	COPD flare	A 67-year-old woman with a history of COPD presents with 3 days of worsening dyspnea and increased frequency of coughing. Her cough is now productive of green, purulent sputum. The patient has a 100-pack-year history of smoking. She has had intermittent, low-grade fever of 100°F (37.7°C) for the past 3 days and her appetite is poor. She has required increased use of rescue bronchodilator therapy in addition to her maintenance medications to control symptoms.	A 67-year-old woman with a history of COPD presents with 3 days of worsening dyspnea and increased frequency of coughing. Her cough is now productive of green, purulent sputum. The patient has a 100-pack-year history of smoking. She has had intermittent, low-grade fever of 100°F (37.7°C) for the past 3 days and her appetite is poor. She has required increased use of rescue bronchodilator therapy in addition to her maintenance medications to control symptoms.	-	-
5	Deep vein thrombosis	A 65-year-old woman presents with unilateral leg pain and swelling of 5 days' duration. There is a history of hypertension, mild CHF, and recent hospitalization for pneumonia. She had been recuperating at home but on beginning to mobilize and walk, the right leg became painful, tender, and swollen. On examination, the right calf is 4 cm greater in circumference than the left when measured 10 cm below the tibial tuberosity. Superficial veins in the leg are more dilated on the right foot and the right leg is slightly redder than the left. There is some tenderness on palpation in the popliteal fossa behind the knee.	A 65-year-old woman presents with unilateral leg pain and swelling of 5 days' duration. There is a history of hypertension, mild CHF, and recent hospitalization for pneumonia. She had been recuperating at home but on beginning to mobilize and walk, the right leg became painful, tender, and swollen.	On examination, the right calf is 4 cm greater in circumference than the left when measured 10 cm below the tibial tuberosity. Superficial veins in the leg are more dilated on the right foot and the right leg is slightly redder than the left. There is some tenderness on palpation in the popliteal fossa behind the knee.	-
6	Heart Attack	Mr. Y is a 64 year old Chinese male who presents with chest pain for 24 hours. One day prior to presentation, the patient began to experience 8/10, non-radiating substernal chest pressure associated with diaphoresis and shortness of breath. The pain intially improved with Tylenol, however over the following 24 hours, his symptoms worsened. The patient went to his primary physician, where an EKG was performed which showed ST elevation in leads V2-V6.	Mr. Y is a 64 year old Chinese male who presents with chest pain for 24 hours. One day prior to presentation, the patient began to experience 8/10, non-radiating substernal chest pressure associated with diaphoresis and shortness of breath. The pain intially improved with Tylenol, however over the following 24 hours, his symptoms worsened.	-	The patient went to his primary physician, where an EKG was performed which showed ST elevation in leads V2-V6.
7	Hemolytic uremic syndrome	A 4-year-old boy presents with a 7-day history of abdominal pain and watery diarrhea that became bloody after the first day. Three days before the onset of symptoms, he had visited the county fair with his family and had eaten a hamburger. Physical examination reveals a mild anemia.	A 4-year-old boy presents with a 7-day history of abdominal pain and watery diarrhea that became bloody after the first day. Three days before the onset of symptoms, he had visited the county fair with his family and had eaten a hamburger.	Physical examination reveals a mild anemia.	-
8	Kidney stones	A 45-year-old white man presents to the emergency department with a 1-hour history of sudden onset of left-sided flank pain radiating down toward his groin. The patient is writhing in pain, which is unrelieved by position. He also complains of nausea and vomiting.	A 45-year-old white man presents to the emergency department with a 1-hour history of sudden onset of left-sided flank pain radiating down toward his groin. The patient is writhing in pain, which is unrelieved by position. He also complains of nausea and vomiting.	-	-
9	Malaria	A 28-year-old man presents to his physician with a 5-day history of fever, chills, and rigors, not improving with acetaminophen (paracetamol), along with diarrhea. He had been traveling in Central America for 3 months, returning 8 weeks ago. He had been bitten by mosquitoes on multiple occasions, and although he initially took malaria prophylaxis, he discontinued it due to mild nausea. He does not know the specifics of his prophylactic therapy. On examination he has a temperature of 100.4°F (38°C), and is mildly tachycardic with a BP of 126/82 mmHg. The remainder of the examination is normal.	A 28-year-old man presents to his physician with a 5-day history of fever, chills, and rigors, not improving with acetaminophen (paracetamol), along with diarrhea. He had been traveling in Central America for 3 months, returning 8 weeks ago. He had been bitten by mosquitoes on multiple occasions, and although he initially took malaria prophylaxis, he discontinued it due to mild nausea. He does not know the specifics of his prophylactic therapy.	On examination he has a temperature of 100.4°F (38°C), and is mildly tachycardic with a BP of 126/82 mmHg. The remainder of the examination is normal.	-
10	Meningitis	An 18-year-old male student presents with severe headache and fever that he has had for 3 days. Examination reveals fever, photophobia, and neck stiffness.	An 18-year-old male student presents with severe headache and fever that he has had for 3 days.	Examination reveals fever, photophobia, and neck stiffness.	-
11	Pneumonia	A 65-year-old man with hypertension and degenerative joint disease presents to the emergency department with a three-day history of a productive cough and fever. He has a temperature of 38.3°C (101°F), a blood pressure of 144/92 mm Hg, a respiratory rate of 22 breaths per minute, a heart rate of 90 beats per minute, and oxygen saturation of 92 percent while breathing room air. Physical examination reveals only crackles and egophony in the right lower lung field. The white-cell count is 14,000 per cubic millimeter, and the results of routine chemical tests are normal. A chest radiograph shows an infiltrate in the right lower lobe.	A 65-year-old man with hypertension and degenerative joint disease presents to the emergency department with a three-day history of a productive cough and fever.	He has a temperature of 38.3°C (101°F), a blood pressure of 144/92 mm Hg, a respiratory rate of 22 breaths per minute, a heart rate of 90 beats per minute, and oxygen saturation of 92 percent while breathing room air. Physical examination reveals only crackles and egophony in the right lower lung field.	The white-cell count is 14,000 per cubic millimeter, and the results of routine chemical tests are normal. A chest radiograph shows an infiltrate in the right lower lobe.
12	Pulmonary embolism	A 65-year-old man presents to the emergency department with acute onset of SOB of 30 minutes' duration. Initially, he felt faint but did not lose consciousness. He is complaining of left-sided chest pain that worsens on deep inspiration. He has no history of cardiopulmonary disease. A week ago he underwent a total left hip replacement and, following discharge, was on bed rest for 3 days due to poorly controlled pain. He subsequently noticed swelling in his left calf, which is tender on examination. His current vital signs reveal a fever of 100.4°F (38.0°C), heart rate 112 bpm, BP 95/65, and an O2 saturation on room air of 91%.	A 65-year-old man presents to the emergency department with acute onset of SOB of 30 minutes' duration. Initially, he felt faint but did not lose consciousness. He is complaining of left-sided chest pain that worsens on deep inspiration. He has no history of cardiopulmonary disease. A week ago he underwent a total left hip replacement and, following discharge, was on bed rest for 3 days due to poorly controlled pain. He subsequently noticed swelling in his left calf, which is tender on examination.	His current vital signs reveal a fever of 100.4°F (38.0°C), heart rate 112 bpm, BP 95/65, and an O2 saturation on room air of 91%.	-
13	Rocky Mountain Spotted Fever	An 8-year-old boy in Oklahoma is brought to the emergency department over the fourth of July weekend because of fever, chills, malaise, athralgias, and a headache. Physical examination reveals a maculopapular rash that is most prominent on his wrists and ankles.	An 8-year-old boy in Oklahoma is brought to the emergency department over the fourth of July weekend because of fever, chills, malaise, athralgias, and a headache.	Physical examination reveals a maculopapular rash that is most prominent on his wrists and ankles.	-
14	Stroke	A 70-year-old man with a history of chronic HTN and atrial fibrillation is witnessed by a family member to have nausea, vomiting, and right-sided weakness, as well as difficulty speaking and comprehending language. The symptoms started with only mild slurred speech before progressing over several minutes to severe aphasia and right arm paralysis. The patient is taking warfarin.	A 70-year-old man with a history of chronic HTN and atrial fibrillation is witnessed by a family member to have nausea, vomiting, and right-sided weakness, as well as difficulty speaking and comprehending language. The symptoms started with only mild slurred speech before progressing over several minutes to severe aphasia and right arm paralysis. The patient is taking warfarin.	-	-
15	Tetanus	A 63-year-old man sustained a cut on his hand while gardening. His immunization history is significant for not having received a complete tetanus immunization schedule. He presents with signs of generalized tetanus with trismus ("lock jaw"), which results in a grimace described as "risus sardonicus" (sardonic smile). Intermittent tonic contraction of his skeletal muscles causes intensely painful spasms, which last for minutes, during which he retains consciousness. The spasms are triggered by external (noise, light, drafts, physical contact) or internal stimuli, and as a result he is at the risk of sustaining fractures or developing rhabdomyolysis. The tetanic spasms also produce opisthotonus, board-like abdominal wall rigidity, dysphagia, and apneic periods due to contraction of the thoracic muscles and/or glottal or pharyngeal muscles. During a generalized spasm the patient arches his back, extends his legs, flexes his arms in abduction, and clenches his fists. Apnea results during some of the spasms. Autonomic overactivity initially manifests as irritability, restlessness, sweating, and tachycardia. Several days later this may present as hyperpyrexia, cardiac arrhythmias, labile hypertension, or hypotension.	A 63-year-old man sustained a cut on his hand while gardening. His immunization history is significant for not having received a complete tetanus immunization schedule. He presents with signs of generalized tetanus with trismus ("lock jaw"), which results in a grimace described as "risus sardonicus" (sardonic smile). Intermittent tonic contraction of his skeletal muscles causes intensely painful spasms, which last for minutes, during which he retains consciousness. The spasms are triggered by external (noise, light, drafts, physical contact) or internal stimuli, and as a result he is at the risk of sustaining fractures or developing rhabdomyolysis. The tetanic spasms also produce opisthotonus, board-like abdominal wall rigidity, dysphagia, and apneic periods due to contraction of the thoracic muscles and/or glottal or pharyngeal muscles. During a generalized spasm the patient arches his back, extends his legs, flexes his arms in abduction, and clenches his fists. Apnea results during some of the spasms. Autonomic overactivity initially manifests as irritability, restlessness, sweating, and tachycardia. Several days later this may present as hyperpyrexia, cardiac arrhythmias, labile hypertension, or hypotension.	-	-
16	Acute otitis media	An 18-month-old toddler presents with 1 week of rhinorrhea, cough, and congestion. Her parents report she is irritable, sleeping restlessly, and not eating well. Overnight she developed a fever. She attends day care and both parents smoke. On examination signs are found consistent with a viral respiratory infection including rhinorrhea and congestion. The toddler appears irritable and apprehensive and has a fever. Otoscopy reveals a bulging, erythematous tympanic membrane and absent landmarks.	An 18-month-old toddler presents with 1 week of rhinorrhea, cough, and congestion. Her parents report she is irritable, sleeping restlessly, and not eating well. Overnight she developed a fever. She attends day care and both parents smoke.	On examination signs are found consistent with a viral respiratory infection including rhinorrhea and congestion. The toddler appears irritable and apprehensive and has a fever.	Otoscopy reveals a bulging, erythematous tympanic membrane and absent landmarks.
17	Acute pharyngitis	A 7-year-old girl presents with abrupt onset of fever, nausea, vomiting, and sore throat. The child denies cough, rhinorrhea, or nasal congestion. On physical exam, oral temperature is 101°F (38.5°C) and there is an exudative pharyngitis, with enlarged cervical lymph nodes. A rapid antigen test is positive for group A Streptococcus (GAS)	A 7-year-old girl presents with abrupt onset of fever, nausea, vomiting, and sore throat. The child denies cough, rhinorrhea, or nasal congestion.	On physical exam, oral temperature is 101°F (38.5°C) and there is an exudative pharyngitis, with enlarged cervical lymph nodes.	A rapid antigen test is positive for group A Streptococcus (GAS)
18	Acute pharyngitis	Mr. A is a 24 year-old man who presents to your office for complaints of sore throat, fever, and headache. His symptoms started 2 days ago with acute onset of sore throat and fever to 102.2. He has had no cough. His physical examination is normal, except for the presence of tonsillar exudates and some tender anterior cervical lymphadenopathy. He is otherwise in good health, and is on no medications except for ibuprofen for fever. He has no drug allergies. (, Centor score = 4 – treat, or test and treat)	Mr. A is a 24 year-old man who presents to your office for complaints of sore throat, fever, and headache. His symptoms started 2 days ago with acute onset of sore throat and fever to 102.2. He has had no cough.<br>He is otherwise in good health, and is on no medications except for ibuprofen for fever. He has no drug allergies. (, Centor score = 4 – treat, or test and treat)	His physical examination is normal, except for the presence of tonsillar exudates and some tender anterior cervical lymphadenopathy.	-
19	Acute sinusitis	Mrs. S is a 35 year-old woman who presents with 15 days of nasal congestion. She has had facial pain and green nasal discharge for the last 12 days. She has had no fever. On physical examination, she has no fever and the only abnormal finding is maxillary tenderness on palpation. She is otherwise healthy, except for mild obesity. She is on no medications, except for an over-the-counter decongestant. She has no drug allergies	Mrs. S is a 35 year-old woman who presents with 15 days of nasal congestion. She has had facial pain and green nasal discharge for the last 12 days. She has had no fever.She is on no medications, except for an over-the-counter decongestant. She has no drug allergies	On physical examination, she has no fever and the only abnormal finding is maxillary tenderness on palpation. She is otherwise healthy, except for mild obesity.	-
20	Back pain	Consider a 35-year-old man who developed low back pain after shoveling snow 3 weeks ago. He presents to the office for an evaluation. On examination there is a new left foot drop. In study 82% physicians recommend MRI (sciatica/sprain)			
21	Cellulitis	A 45-year-old man presents with acute onset of pain and redness of the skin of his lower extremity. Low-grade fever is present and the pretibial area is erythematous, edematous, and tender.	A 45-year-old man presents with acute onset of pain and redness of the skin of his lower extremity.	Low-grade fever is present and the pretibial area is erythematous, edematous, and tender.	-
22	COPD flare (milder)	A 56-year-old woman with a history of smoking presents to her primary care physician with shortness of breath and cough for several days. Her symptoms began 3 days ago with rhinorrhea. She reports a chronic morning cough productive of white sputum, which has increased over the past 2 days. She has had similar episodes each winter for the past 4 years. She has smoked 1 to 2 packs of cigarettes per day for 40 years and continues to smoke. She denies hemoptysis, chills, or weight loss and has not received any relief from over-the-counter cough preparations.	A 56-year-old woman with a history of smoking presents to her primary care physician with shortness of breath and cough for several days. Her symptoms began 3 days ago with rhinorrhea. She reports a chronic morning cough productive of white sputum, which has increased over the past 2 days. She has had similar episodes each winter for the past 4 years. She has smoked 1 to 2 packs of cigarettes per day for 40 years and continues to smoke. She denies hemoptysis, chills, or weight loss and has not received any relief from over-the-counter cough preparations.	-	-
23	Influenza	A 30-year-old woman presents in January with 2-day history of fever, cough, headache, and generalized weakness. She was in her usual state of health before an abrupt onset of these symptoms. A few viral illnesses have affected her during the current winter, but not to this severity. She reports sick contacts at work and did not receive the seasonal influenza vaccine this season.	A 30-year-old woman presents in January with 2-day history of fever, cough, headache, and generalized weakness. She was in her usual state of health before an abrupt onset of these symptoms. A few viral illnesses have affected her during the current winter, but not to this severity. She reports sick contacts at work and did not receive the seasonal influenza vaccine this season.	-	-
24	Mononucleosis	A 16-year-old female high school student presents with complaints of fever, sore throat, and fatigue. She started feeling sick 1 week ago. Her symptoms are gradually getting worse, and she has difficulty swallowing. She has had a fever every day, and she could hardly get out of bed this morning. She does not remember being exposed to anybody with a similar illness recently. On physical examination she is febrile and looks sick. Enlarged cervical lymph nodes, exudative pharyngitis with soft palate petechiae and faint erythematous macular rash on the trunk and arms are found.	A 16-year-old female high school student presents with complaints of fever, sore throat, and fatigue. She started feeling sick 1 week ago. Her symptoms are gradually getting worse, and she has difficulty swallowing. She has had a fever every day, and she could hardly get out of bed this morning. She does not remember being exposed to anybody with a similar illness recently.	On physical examination she is febrile and looks sick. Enlarged cervical lymph nodes, exudative pharyngitis with soft palate petechiae and faint erythematous macular rash on the trunk and arms are found.	-
25	Peptic Ulcer Disease	A 40-year-old man presents to his primary care physician with a 2-month history of intermittent upper abdominal pain. He describes the pain as a dull, gnawing ache. The pain sometimes wakes him at night, is relieved by food and drinking milk, and is helped partially by ranitidine. He had a similar but milder episode about 5 years ago, which was treated with omeprazole. Physical examination reveals a fit, apparently healthy man in no distress. The only abnormal finding is mild epigastric tenderness on palpation of the abdomen.	A 40-year-old man presents to his primary care physician with a 2-month history of intermittent upper abdominal pain. He describes the pain as a dull, gnawing ache. The pain sometimes wakes him at night, is relieved by food and drinking milk, and is helped partially by ranitidine. He had a similar but milder episode about 5 years ago, which was treated with omeprazole.	Physical examination reveals a fit, apparently healthy man in no distress. The only abnormal finding is mild epigastric tenderness on palpation of the abdomen.	-
26	Pneumonia	A 6-year-old boy with a medical history significant for mild persistent asthma is brought to the clinic by his mother with a history of a 5-day cough. His mother reports that the child's fever continues to be elevated despite acetaminophen therapy. He has missed school for the past 3 days and he has a classmate sick with pneumonia. The mother reports that the appetite is good for the child. His cough produced yellowish sputum at home. His vitals at the clinic are: respiratory rate 19 breaths/min, heart rate 80 beats/min, and temperature 101.6°F (38.7°C). He appears in no respiratory distress. His lung examination reveals bilateral rales and occasional wheeze. CXR reveals lobar infiltrates without pleural effusions.	A 6-year-old boy with a medical history significant for mild persistent asthma is brought to the clinic by his mother with a history of a 5-day cough. His mother reports that the child's fever continues to be elevated despite acetaminophen therapy. He has missed school for the past 3 days and he has a classmate sick with pneumonia. The mother reports that the appetite is good for the child. His cough produced yellowish sputum at home.	His vitals at the clinic are: respiratory rate 19 breaths/min, heart rate 80 beats/min, and temperature 101.6°F (38.7°C). He appears in no respiratory distress. His lung examination reveals bilateral rales and occasional wheeze.	CXR reveals lobar infiltrates without pleural effusions.
27	Salmonella	A 14-year-old boy presents with nausea, vomiting, and diarrhea. Eighteen hours earlier, he had been at a picnic where he ingested undercooked chicken along with a variety of other foods. He reports moderate-volume, nonbloody stools occurring 6 times a day. He has mild abdominal cramps and a low-grade fever. He is evaluated at an acute care clinic and found to be mildly tachycardic (heart rate 105 bpm) with a normal BP and a low-grade temperature of 100.1°F (37.8°C). His physical exam is unremarkable except for mild diffuse abdominal tenderness and mild increased bowel sounds. He is able to take oral fluids and is instructed on the appropriate oral fluid and electrolyte rehydration.	A 14-year-old boy presents with nausea, vomiting, and diarrhea. Eighteen hours earlier, he had been at a picnic where he ingested undercooked chicken along with a variety of other foods. He reports moderate-volume, nonbloody stools occurring 6 times a day. He has mild abdominal cramps and a low-grade fever.	He is evaluated at an acute care clinic and found to be mildly tachycardic (heart rate 105 bpm) with a normal BP and a low-grade temperature of 100.1°F (37.8°C). His physical exam is unremarkable except for mild diffuse abdominal tenderness and mild increased bowel sounds.	-
28	Shingles	A 77-year-old man reports a 5-day history of burning and aching pain on the right side of his chest. This is followed by the development of erythema and a maculopapular rash in this painful area, accompanied by headache and malaise. The rash progressed to develop clusters of clear vesicles for 3 to 5 days, evolving through stages of pustulation, ulceration, and crusting.	A 77-year-old man reports a 5-day history of burning and aching pain on the right side of his chest. This is followed by the development of erythema and a maculopapular rash in this painful area, accompanied by headache and malaise. The rash progressed to develop clusters of clear vesicles for 3 to 5 days, evolving through stages of pustulation, ulceration, and crusting.	-	-
29	Urinary tract infection	A 26-year-old female newly wed presents complaining of painful urination, feeling of urgent need to urinate, and more frequent urination for 2 days. She denies any fever, chills, nausea, vomiting, back pain, vaginal discharge, or vaginal pruritus.	A 26-year-old female newly wed presents complaining of painful urination, feeling of urgent need to urinate, and more frequent urination for 2 days. She denies any fever, chills, nausea, vomiting, back pain, vaginal discharge, or vaginal pruritus.	-	-
30	Vertigo	A 65-year-old woman presents with a chief complaint of dizziness. She describes it as a sudden and severe spinning sensation precipitated by rolling over in bed onto her right side. Symptoms typically last <30 seconds. They have occurred nightly over the last month and occasionally during the day when she tilts her head back to look upward. She describes no precipitating event prior to onset and no associated hearing loss, tinnitus, or other neurologic symptoms. Otologic and neurologic examinations are normal except for the Dix-Hallpike maneuver, which is negative on the left but strongly positive on the right side.	A 65-year-old woman presents with a chief complaint of dizziness. She describes it as a sudden and severe spinning sensation precipitated by rolling over in bed onto her right side. Symptoms typically last <30 seconds. They have occurred nightly over the last month and occasionally during the day when she tilts her head back to look upward. She describes no precipitating event prior to onset and no associated hearing loss, tinnitus, or other neurologic symptoms.	Otologic and neurologic examinations are normal except for the Dix-Hallpike maneuver, which is negative on the left but strongly positive on the right side.	-
31	Acute bronchitis	A 34-year-old woman with no known underlying lung disease 12-day history of cough. She initially had nasal congestion and a mild sore throat, but now her symptoms are all related to a productive cough without paroxysms. She denies any sick contacts. On physical examination she is not in respiratory distress and is afebrile with normal vital signs. No signs of URI are noted. Scattered wheezes are present diffusely on lung auscultation.	A 34-year-old woman with no known underlying lung disease 12-day history of cough. She initially had nasal congestion and a mild sore throat, but now her symptoms are all related to a productive cough without paroxysms. She denies any sick contacts.	On physical examination she is not in respiratory distress and is afebrile with normal vital signs. No signs of URI are noted. Scattered wheezes are present diffusely on lung auscultation.	-
32	Acute bronchitis	Mrs. L is a 61 year-old woman who presents with 4 days of a cough productive of yellow sputum. Her symptoms started 4 days ago with rhinorrhea and productive cough. She initially had fevers as high as 101 for 2 days, but those have now resolved. In the office, she has normal vital signs and a normal physical examination. She is otherwise healthy except for high cholesterol for which she is being treated with atorvastatin. She has no drug allergies.	Mrs. L is a 61 year-old woman who presents with 4 days of a cough productive of yellow sputum. Her symptoms started 4 days ago with rhinorrhea and productive cough. She initially had fevers as high as 101 for 2 days, but those have now resolved.She is otherwise healthy except for high cholesterol for which she is being treated with atorvastatin. She has no drug allergies.	In the office, she has normal vital signs and a normal physical examination.	-
33	Acute conjunctivitis	A 14-year-old boy with no significant past medical history presents 3 days after developing a red, irritated right eye thatspreadtothelefteyetoday.Hehaswaterydischargefrombotheyesandtheyarestuckshutinthemorning.He reports recent upper respiratory symptoms and that several children at his day camp recently had pink eye. He denies significant pain or light sensitivity and does not wear contact lenses. On examination, his pupils are equal and reactive and he has a right-sided, tender preauricular lymph node. Penlight examination does not reveal any corneal opacity.	A 14-year-old boy with no significant past medical history presents 3 days after developing a red, irritated right eye thatspreadtothelefteyetoday.Hehaswaterydischargefrombotheyesandtheyarestuckshutinthemorning.He reports recent upper respiratory symptoms and that several children at his day camp recently had pink eye. He denies significant pain or light sensitivity and does not wear contact lenses.	On examination, his pupils are equal and reactive and he has a right-sided, tender preauricular lymph node. Penlight examination does not reveal any corneal opacity.	-
34	Acute pharyngitis	Mr. E is a 26 year-old man who presents to your office for complaints of sore throat, headache, and non-productive cough. His symptoms started 2 days ago with acute onset of sore throat. He has been afebrile. His physical examination is normal, except for some pharyngeal erythema. He is otherwise in good health, and is on no medications except for acetaminophen for his sore throat and fever. He has no drug **allergies.s**	Mr. E is a 26 year-old man who presents to your office for complaints of sore throat, headache, and non-productive cough. His symptoms started 2 days ago with acute onset of sore throat. He has been afebrile. He is otherwise in good health, and is on no medications except for acetaminophen for his sore throat and fever. He has no drug **allergies.s**	His physical examination is normal, except for some pharyngeal erythema.	-
35	Allergic rhinitis	A 22-year-old student presents with a 5-year history of worsening nasal congestion, sneezing, and nasal itching. Symptoms are year-round but worse during the spring season. On further questioning it is revealed that he has significant eye itching, redness, and tearing as well as palate and throat itching during the spring season. He remembers that his mother told him at some point that he used to have eczema in infancy.	A 22-year-old student presents with a 5-year history of worsening nasal congestion, sneezing, and nasal itching. Symptoms are year-round but worse during the spring season. On further questioning it is revealed that he has significant eye itching, redness, and tearing as well as palate and throat itching during the spring season. He remembers that his mother told him at some point that he used to have eczema in infancy.	-	-
36	Back pain	A 38-year-old man with no significant history of back pain developed acute LBP when lifting boxes 2 weeks ago. The pain is aching in nature, located in the left lumbar area, and associated with spasms. He describes previous similar episodes several years ago, which resolved without seeing a doctor. He denies any leg pain or weakness. He also denies fevers, chills, weight loss, and recent infections. Over-the-counter ibuprofen has helped somewhat, but he has taken it only twice a day for the past 3 days because he does not want to become dependent on painkillers. On examination, there is decreased lumbar flexion and extension secondary to pain, but a neurologic exam is unremarkable.			
37	Bee sting without anaphylaxis	A 9-year-old boy is brought to the ER after being stung by a bee at a picnic. He is crying hysterically. After 15 minutes of calming him down, exam reveals a swollen tender upper lip but no tongue swelling, no drooling, no stridor, no rash, and no other complaints.	A 9-year-old boy is brought to the ER after being stung by a bee at a picnic. He is crying hysterically.	After 15 minutes of calming him down, exam reveals a swollen tender upper lip but no tongue swelling, no drooling, no stridor, no rash, and no other complaints.	-
38	Canker sore	A 17-year-old male student presents with recurrent mouth ulceration since his early schooldays. He has no respiratory, anogenital, gastrointestinal, eye, or skin lesions. His mother had a similar history as a teenager. The social history includes no tobacco use and virtually no alcohol consumption. He has no history of recent drug or medication ingestion. Extraoral exam reveals no significant abnormalities and specifically no pyrexia; no cervical lymph node enlargement; nor cranial nerve, salivary, or temporomandibular joint abnormalities. Oral exam reveals a well-restored dentition and there is no clinical evidence of periodontal-attachment loss or pocketing. He has five 4 mm round ulcers with inflammatory haloes in his buccal mucosae.	A 17-year-old male student presents with recurrent mouth ulceration since his early schooldays. He has no respiratory, anogenital, gastrointestinal, eye, or skin lesions. His mother had a similar history as a teenager. The social history includes no tobacco use and virtually no alcohol consumption. He has no history of recent drug or medication ingestion.	Extraoral exam reveals no significant abnormalities and specifically no pyrexia; no cervical lymph node enlargement; nor cranial nerve, salivary, or temporomandibular joint abnormalities. Oral exam reveals a well-restored dentition and there is no clinical evidence of periodontal-attachment loss or pocketing. He has five 4 mm round ulcers with inflammatory haloes in his buccal mucosae.	-
39	Candidal yeast infection	Consider a 40-year-old, monogamous, married woman who calls to report a 2-day history of vaginal itching and thick white discharge. She has no abdominal pain or fever. (In study 50% recommended physician visit)	Consider a 40-year-old, monogamous, married woman who calls to report a 2-day history of vaginal itching and thick white discharge.	She has no abdominal pain or fever. (In study 50% recommended physician visit)	-
40	Constipation	A 5-month-old baby boy presents with difficulty and delay in passing hard stools. His mother reports that he strains for several hours and may even miss a day, before passing stool with screaming and occasional spots of fresh blood on the stool or diaper. He has recently been weaned from breastfeeding to cows' milk formula, which he had been reluctant to drink initially. The child is thriving and now feeding normally. There was no neonatal delay in defecation and no history of excessive vomiting or abdominal distension.			
41	Eczema	A 12-year-old female presents with dry, itchy skin that involves the flexures in front of her elbows, behind her knees, and in front of her ankles. Her cheeks also have patches of dry, scaly skin. She has symptoms of hay fever and has recently been diagnosed with egg and milk allergy. She has a brother with asthma and an uncle and several cousins who have been diagnosed with eczema.	A 12-year-old female presents with dry, itchy skin that involves the flexures in front of her elbows, behind her knees, and in front of her ankles. Her cheeks also have patches of dry, scaly skin. She has symptoms of hay fever and has recently been diagnosed with egg and milk allergy. She has a brother with asthma and an uncle and several cousins who have been diagnosed with eczema.	-	-
42	Stye	A 30-year-old man presents with a painful, swollen right eye for the past day. He reports minor pain on palpation of the eyelid and denies any history of trauma, crusting, or change in vision. He has no history of allergies or any eye conditions and denies the use of any new soaps, lotions, or creams. On exam, he has localized tenderness to palpation and erythema on the midline of the lower eyelid near the lid margin. The remainder of the physical exam, including the globe, is normal.	A 30-year-old man presents with a painful, swollen right eye for the past day. He reports minor pain on palpation of the eyelid and denies any history of trauma, crusting, or change in vision. He has no history of allergies or any eye conditions and denies the use of any new soaps, lotions, or creams.	On exam, he has localized tenderness to palpation and erythema on the midline of the lower eyelid near the lid margin. The remainder of the physical exam, including the globe, is normal.	-
43	Viral upper respiratory	Mr. R. is a 56 year-old man who presents to you with 6 days of non-productive cough, nasal congestion, and green nasal discharge. He has had intermittent fevers as high as 100.8. His physical examination is normal except for rhinorrhea. He is otherwise healthy, except for chronic osteoarthritis of the right knee. He has no drug allergies.	Mr. R. is a 56 year-old man who presents to you with 6 days of non-productive cough, nasal congestion, and green nasal discharge.He has had intermittent fevers as high as 100.8.He has no drug allergies.	His physical examination is normal except for rhinorrhea. He is otherwise healthy, except for chronic osteoarthritis of the right knee.	-
44	Viral upper respiratory	A 30-year-old man presents with a 2-day history of runny nose and sore throat. He feels hot and sweaty, has a mild headache, is coughing up clear sputum and complains of muscle aches. He would like antibiotics as he was prescribed them last year when he had a similar condition. On examination, he is afebrile, has a normal pulse, a slightly inflamed pharynx and nontender cervical lymphadenopathy. There is no neck stiffness and his chest is clear. He has tried over-the-counter cough medications, but has not found these helpful. He smokes 10 cigarettes per day.	A 30-year-old man presents with a 2-day history of runny nose and sore throat. He feels hot and sweaty, has a mild headache, is coughing up clear sputum and complains of muscle aches. He would like antibiotics as he was prescribed them last year when he had a similar condition. He has tried over-the-counter cough medications, but has not found these helpful. He smokes 10 cigarettes per day.	On examination, he is afebrile, has a normal pulse, a slightly inflamed pharynx and nontender cervical lymphadenopathy. There is no neck stiffness and his chest is clear.	-
45	Vomiting	Elizabeth’s 2-year-old son has a fever and vomited twice. Elizabeth worries about dehydration, so she gives Jack a sippy cup of apple juice. He immediately vomits up the juice. Elizabeth debates what to do next. Should she try to reach Jack’s pediatrician or should she take Jack to the ED? Instead, she calls her triage nurse line. Temperature = 100.5			
